# Translating research flow cytometry assays for characterization of cellular therapeutics

**DOI:** 10.1186/1476-9255-12-S1-O8

**Published:** 2015-04-16

**Authors:** John DM Campbell

**Affiliations:** 1 Research, Development & Innovation, Scottish National Blood Transfusion Service (SNBTS), Edinburgh EH17 7QT, UK

## 

Cellular therapy is a rapidly growing area of medicine, particularly immune therapies for infection and cancer, as well as therapies that aim to regenerate diseased or damaged tissues. Flow cytometry is one of the most important tools in the development of novel cellular therapeutics. Cellular identity, viability and differentiation status are essential data to be collected and validated throughout the manufacturing process. Flow cytometry is used in characterization of raw starting material; as an in-process control; to define the final product and also is often an essential tool to monitor responses in treated patients. In this presentation, the technical aspects of whole blood rare cell flow cytometry (such as circulating dendritic cells) were discussed, and how this can be applied to complex cultures of stem cells differentiating into blood cells for therapeutic use. These cultures contain CD45+ stem cells; nucleated RBC; enucleated RBC; as well as free nuclei and debris. Enucleating *in vitro* RBC could be simply quantified by using staining with CD45, CD235a, DRAQ5 DNA staining and DAPI dead cell exclusion. Staining was performed on raw culture supernatant (as would be performed in a no lyse no wash blood preparation), then diluted before flow cytometry analysis. The other area discussed in this presentation was the improved identification of chemokine receptor expression on cells for therapy by flow cytometry. Chemokines orchestrate the movement and homing of cells in the body. The ability to manipulate the expression of chemokine receptors is a major focus of cellular therapeutics research. In this presentation, the use of biotinylated chemokines tetramerized with streptavidin-fluorochromes was discussed as a method to improve staining of chemokine receptors by flow cytometry. These labelled human ligands stain cells strongly, often more strongly than antibodies, and have the unique advantage that they can be used across species boundaries. Additionally, these reagents can be combined with magnetic bead sorting to isolate chemokine receptor-positive cells with improved *in vitro* and *in vivo* migration capacity [1].
